# Indicator bacteria in beaver ponds—research from Poland

**DOI:** 10.1128/spectrum.01092-25

**Published:** 2025-11-17

**Authors:** Michał Wróbel, Radosław Gawryś, Magdalena Frąk, Anna Tereba, Andrzej Boczoń, Tomasz Wojda

**Affiliations:** 1 Department of Forest Ecology, Forest Research Institute, Sękocin Stary, Poland; 2 Department of Remote Sensing and Environmental Assessment, Warsaw University of Life Sciences 49561, Warsaw, Poland; 3 Department of Silviculture and Genetics of Forest Trees, Forest Research Institute, Sękocin Stary, Poland; Connecticut Agricultural Experiment Station, New Haven, Connecticut, USA

**Keywords:** European beaver (Castor fiber L.), beaver ponds, bacteria, 16S rRNA sequencing

## Abstract

Beaver ponds create unique aquatic ecosystems. The activities of beavers promote the growth of biodiversity and enhance water purification but can also lead to the accumulation of harmful pollutants. Water retention in beaver ponds leads to the growth of bacteria and other microorganisms that play a key role in biogeochemical cycling. Stagnation of water can lead to anaerobic conditions and the formation of toxic substances that can limit benthic diversity. Beavers play an important role in shaping these environments, and the study of bacteria in beaver ponds helps to better understand their impact on aquatic ecosystems. A 16S rRNA sequencing revealed the presence of 365 bacterial species in water and sediments, identified in 174 genera and 83 families, 62 of which are present in water and sediments. The main indicator family for the bacteria identified in water samples was the family *Xantonomodaceae*, while for the sediments, it was the family *Planococcaceae*. The occurrence of bacterial families was similar in both substrates, but sediments showed greater variability compared to water. The statistical differences in the distribution of families between water and sediments were confirmed by a paired sample T test.

**IMPORTANCE:**

This study helps us to understand how beavers shape their environment and influence water quality. By building dams, beavers create wetlands that change the flow of water and influence which bacteria thrive there. Some of these bacteria contribute to the natural purification of water, while others can pose a threat to biodiversity. Knowing how the activities of beavers affect microbial life is important to protect ecosystems and ensure water safety. The results can help scientists, conservationists, and policy makers make better decisions about beaver conservation and water management. By understanding the role of bacteria in these wetlands, we can predict both the benefits—such as improved natural filtration and potential risks—such as the spread of harmful bacteria. This research also provides insights into how nature itself filters and purifies water, which could lead to sustainable water management strategies in the future.

## INTRODUCTION

Beaver ponds are unique aquatic ecosystems. Beavers create dams that retain water and transform streams and rivers into floodplains. These transformations affect hydrological, chemical, and biological conditions, altering the microbiological composition of water bodies, including the bacteria present in such reservoirs. It is widely believed that water retention through the activities of the European beaver not only contributes to increasing biodiversity but is also an important element in the water purification process (1–7). However, the long-term accumulation of organic material can also contribute to the accumulation of pollutants. It is therefore not possible to clearly assess the impact of surface water retention by beavers on the intensity of microbiological processes in water and bottom sediments (8).

Aquatic forest ecosystems can contain not only compounds of anthropogenic origin, but also those resulting from natural processes in the catchment area. The main plant substances that flow in large quantities have an effect on increasing the saprobicity of the ecosystem and creating potential anaerobic conditions under conditions of water stagnation (9, 10). This can result in biodegradation processes dominating the ecosystem and the formation of compounds that are toxic to higher organisms, especially in the benthic zone. In addition, beaver dams significantly increase the activity of microorganisms along watercourses, potentially leading to changes in biogeochemical cycles that are directly or indirectly regulated by microorganisms (11).

Beaver dams cause damming of streams, which leads to the formation of pools of stagnant water and can promote the growth of various bacterial species. The slowing of the water flow increases the deposition of suspended solids and organic matter, which creates a rich environment for the growth of microorganisms. Microorganisms that decompose organic matter play a key role in biogeochemical cycles. The colossal aquatic microbial diversity poses a great challenge to understand microbial community dynamics and ecosystem (12). In beaver ponds, their presence is particularly conspicuous due to the abundance of organic material derived from plants and animal waste.

Beaver ponds, especially where the water is low in oxygen, can promote the development of denitrifying bacteria that convert nitrates into gaseous nitrogen, which can lead to a reduction in the concentration of available nitrogen forms in the water. Abundant ammonifying and sulfite-reducing bacteria can cause the accumulation of ammonia and hydrogen sulfide, respectively, in concentrations that are potentially lethal to invertebrate fauna. The consequence of this phenomenon can therefore lead to a reduction in the diversity of benthic communities (2, 9, 10). This phenomenon seems more likely in the case of reservoirs used by beavers during several years of stagnant water.

Beaver ponds often turn into wetlands that can act as natural sewage treatment plants. Microorganisms in such environments help to break down organic and inorganic pollutants. Studies of bacteria in beaver ponds provide a better understanding of the effects of these organisms on local aquatic ecosystems, including processes related to water purification and changes in microbial composition. Tracking changes in bacterial populations can help monitor water quality in beaver ponds and identify potential threats related to biological pollution. Beavers play a key role in shaping aquatic environments, and bacteria are an important element of these ecosystems as they influence both water quality and the functioning of the entire ecosystem. Since only a small percentage of these communities can be identified using classical culture methods (13, 14), a 16S rRNA sequencing approach was used, which offers great potential for studying the dynamics of microbial communities in aquatic ecosystems (12, 15–17).

The manuscript presents insights that may enhance scientific understanding and stimulate further research into the ecological impacts in beaver ponds on habitat conditions.

## MATERIALS AND METHODS

**Fig 1:**
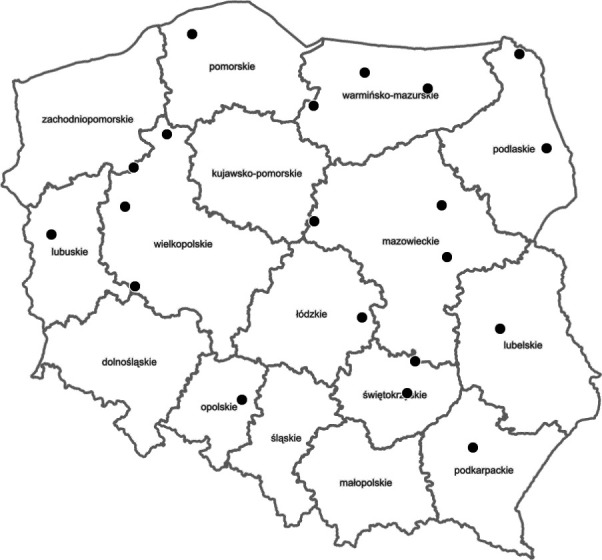
Map of the distribution of research areas against the background of the administrative division of Poland (voivodeships). The map was created using QGIS.

The study was carried out at 20 sites in forest areas throughout Poland (Fig. 1). The site selection depended on the age of the beaver structure, water depth in the pond, thickness of the sediments, width of the valley and reservoir, flow velocity, degree of meandering of the stream, inclination of the valley slopes, and diversity of the forest habitat. The study focused on still water bodies along streams created by the damming of water by beavers. Each selected site had a dam built by beavers that was at least 7 years old. The age of the structure guaranteed long-term maintenance of the impoundment and thus long-term accumulation of sediments.

The molecular analysis of the bacterial populations was performed based on the hypervariable V3–V4 region of the 16S rRNA gene. The use of 16S rRNA sequences has been proposed as a fundamental tool in taxonomic studies of microorganisms (Woese 1997; Johnson et al. 2019). The specific primer sequences 341F: CCTACGGGNGGCWGCAG and 785R: GACTACHVGGGTATCTAATCC (16S analysis) were used to amplify the selected region and prepare the library. PCR amplification was performed using the Q5 Hot Start High-Fidelity 2X Master Mix under the following conditions: for the first PCR (primers 341F and 785R with Nextera adapters), 15 ng of DNA was used. The initial denaturation step was carried out at 98°C for 30 s, followed by 25 cycles of denaturation at 98°C for 10 s, primer annealing at 55°C for 30 s, and elongation at 72°C for 20 s. A final elongation step was performed at 72°C for 2 min, and the reaction was held at 4°C. For the second PCR (Nextera XT indexing), 10 ng of DNA was used. The initial denaturation was also conducted at 98°C for 30 s, followed by 7 cycles of denaturation at 98°C for 10 s, primer annealing at 65°C for 30 s, and elongation at 72°C for 20 s. The final elongation step was performed at 72°C for 2 min, with the reaction held at 4°C. Sequencing was performed on a MiSeq instrument with Paired-End (PE) technology, 2 × 300 nt, using the Illumina v3 kit. Automated preliminary data analysis was performed on a MiSeq sequencer using MiSeq Reporter (MSR) v2.6 software. Bioinformatic analysis, ensuring classification of reads to species level, was performed using the QIIME 2 ver. 2023.9 software package (18) based on the Silva 138 reference sequence database. In addition, the DADA2 package was used to distinguish sequences of biological origin from those that were newly generated during the sequencing process. This package was also used to extract unique sequences of biological origin, i.e., amplicon sequence variants (ASVs).

Molecular tests were performed on all 40 samples collected, 20 of which were collected from bottom sediments and twenty from surface waters. Environmental samples were subjected to the DNA nucleic acid isolation procedure using a commercial kit dedicated to environmental eDNA extraction (Sigma Aldrich, USA). DNA isolation was performed according to the recommended procedure. The final DNA elution was performed in a volume of 100 µL of elution buffer, then the isolates were stored at −20°C until further analysis stages.

For the statistical analyses, identified bacterial families were selected. Statistical analyses were performed using R (version 4.3.1). The IndVal index (19) was used to define specific bacterial groups for a substrate (sediment or water) using the “indicators” function from the “indicspecies” package (20). It is calculated based on the proportion of individuals of given families in a given group in relation to the total and the proportion of sites with given families in a given group. Family-level bacterial diversity between the substrates analyzed was represented using NMDS analysis (“metaMDS” function, “vegan” package, distance euclidean). Only families with a frequency of >5% were included in the NMDS analysis. The significance of differences between species richness, expressed by the Shannon-Wiener diversity index, and mean families relative abundances was compared with the paired sample *t*-test at *P* < 0.05 using the package “stats” (21). Results were considered statistically significant at *P* < 0.05.

## RESULTS

**Fig 2:**
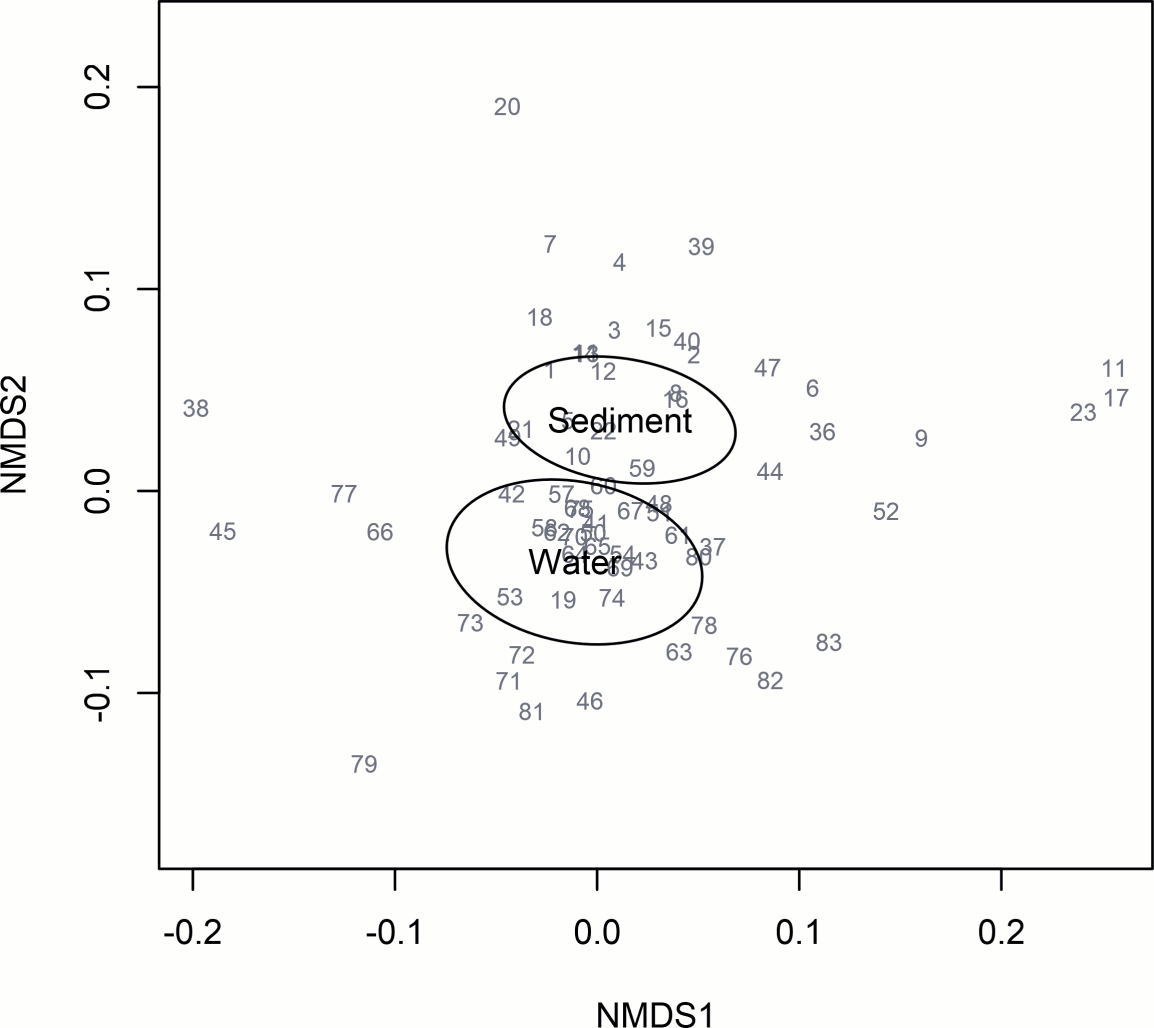
The result of the NMDS analysis shows the diversity of the water and sediment samples (ellipse = 1 SD from centroid) in terms of the bacterial families present (stress = 0.20 at k = 2). The gray color indicates the family numbers, which correspond to the numbering in the table in supplemental material. Families with an occurrence of >5% were considered.

The analysis revealed a total of 365 bacterial species in water and sediment samples, distributed across 174 genera, 83 families, 36 orders, 12 classes, and 8 phyla. The genetic analyses carried out focused on bacterial families, as these could be identified quite well. The family level was chosen for the calculations due to its accuracy of identification. At the next level, the genus was identified as about 70% of the bacteria in some samples. In the collected samples, 83 bacteria families were found, 79 in the sediment samples, and 66 in the water samples. A total of 62 families were found in both substrates. The number of occurrences in the samples and mean number of relative abundance of bacterial families are shown in supplemental material, and the similarities between substrates in terms of the number of individual families are shown in Fig. 2. The most abundant bacterial families in both media were those of *Aeromonadaceae, Pseudomonadaceae, Dysgonomonadaceae, Clostridiaceae,* and *Bacteroidaceae*. In the water samples, the bacterial family *Pseudomonadaceae* dominated in numbers at 13 sampling sites, the bacterial family *Comamonadaceae* in the next three sampling sites and the families *Chromobacteriaceae* and *Aquaspirillaceae* in two sampling sites. In water samples, the *Pseudomonadaceae* and *Comamonadaceae* families had the highest average numbers.

In the sediment samples, the bacterial family *Pseudomonadaceae* had the highest relative abundance (10 sampling sites), similar to the water samples. The second bacterial family with the highest number was *Planococcaceae* (six sampling sites). In three sampling sites, the *Comamonadaceae* family had the largest proportion, and on one surface, the *Chromobacteriaceae* family. In the sediment samples, the families *Pseudomonadaceae*, *Planococcaceae,* and *Comamonadaceae* also had the highest average relative abundances.

A significant difference in mean relative abundance based on paired samples *t*-test at *P* < 0.05 (Table 1) was found in eight families: *Caulobacteraceae, Fusobacteriaceae, Lachnospiraceae, Leptotrichiaceae, Moraxellaceae, Paenibacillaceae, Sporomusaceae, Sutterellaceae,* and one unspecified—family6. The list of families that are indicator taxa for a particular substrate type is shown in Table 2.

**TABLE 1:** Diversity of family composition in water and sediment samples in terms of occurrence and mean number of relative abundance^*d*^

Family	Sediment			Water		
	N^*a*^	M^*b*^	SE^*c*^	N^*a*^	M^*b*^	SE^*c*^
*Caulobacteraceae*	4	0.0046	0.0022	12	0.0495	0.0150
*Family6*	4	0.0087	0.0047	10	0.0818	0.0304
*Fusobacteriaceae*	11	1.0507	0.4882	17	6.9935	1.7621
*Lachnospiraceae*	18	1.1408	0.2039	13	0.2732	0.1268
*Leptotrichiaceae*	2	0.0019	0.0013	9	0.0112	0.0035
*Moraxellaceae*	4	0.0349	0.0217	12	1.0165	0.4630
*Paenibacillaceae*	18	0.3648	0.0599	16	0.1257	0.0322
*Sporomusaceae*	15	0.5437	0.4190	9	0.1005	0.0404
*Sutterellaceae*	8	0.0587	0.0268	.^*e*^	.	.

^*a*^ Number of occurrence.

^*b*^ Arithmetic mean.

^*c*^ Standard error.

^*d*^ Mean values that differ significantly at *P* < 0.05 according to the paired-samples *t*-test.

^*e*^ "." indicates not found in the tested sample.

**TABLE 2:** List of indicator families for water and sediments based on the IndVal index^*c*^

Family	A^*a*^	B^*b*^	IndVal	*P*-value
Water				
*Xanthomonadaceae*	0.99	0.89	0.94	0.005
*Fusobacteriaceae*	0.86	0.84	0.85	0.010
*Moraxellaceae*	0.97	0.58	0.75	0.005
*Enterococcaceae*	0.87	0.68	0.77	0.025
*Caulobacteraceae*	0.90	0.63	0.75	0.005
Sediments				
*Planococcaceae*	0.86	1.00	0.92	0.005
*Desulfovibrionaceae*	0.83	0.58	0.69	0.04
*ST-12K33*	0.99	0.47	0.69	0.005
*Oscillospiraceae*	0.89	0.47	0.65	0.01
*Sutterellaceae*	1.00	0.42	0.65	0.005

^*a*^ Specificity.

^*b*^ Sensivitity.

^*c*^ The table contains only statistically significant results (*P* < 0.05) based on the permutation test with 999 iterations.

The average number of bacterial families found in the water and sediments was 27.3 (SE = 1.67) and 27.8 (SE = 1.21), respectively, while the Shannon-Wiener diversity index was 1.95 (SE = 0.10) and 1.95 (SE = 0.06), respectively. These two traits were not statistically significantly different between substrates based on the paired-samples *t*-test at *P* < 0.05.

## DISCUSSION

Bacteria play a key role in aquatic ecosystems. They are involved in the decomposition and cycling of organic matter (22). This study presents indicator bacterial families for water and sediments of beaver ponds. The studies and statistical analyses carried out have shown that the most important indicator bacteria family identified in water samples is the *Xantonomodaceae* family. The family is widely distributed in the natural environment and individual taxa are a natural component of the soil and water microbiota (23). They can occur in various aquatic environments, such as lakes, rivers, and streams (24). They are particularly common in waters with varying levels of organic matter. They are difficult to identify by culture methods, and molecular studies are revealing that they are present and abundant across different ecosystems. Some *Xanthomonadaceae* species utilize organic matter present in water, which can affect the biological composition of water and biogeochemical cycling in the aquatic ecosystem (25). If these species are also pathogenic to aquatic plants, they can cause changes in the population structure of aquatic plants and affect the stability of the ecosystem. Other families that can be considered as indicators, albeit to a lesser extent, are *Fusobacteriaceae* and *Moraxellaceae*.

In aquatic ecosystems, bacteria of the *Fusobacteriaceae* and *Moraxellaceae* families play an important role in the metabolism of organic substances and in biogeochemical cycles, particularly in the carbon and nitrogen cycles. Representatives of the *Fusobacteriaceae* family are strictly anaerobic bacteria that are mainly found in bottom sediments, anoxic zones, and in waters heavily polluted with organic matter. They play an important role in the mineralization of organic matter and in maintaining the redox balance in anoxic environments. A high number of these bacteria in the sediments can also be an indicator of eutrophication and the deterioration of the ecological status of an aquatic environment. Bacteria from the *Moraxellaceae* family, on the other hand, are aerobic or facultative anaerobes that are frequently found in surface waters and reservoirs. They play an important role in the decomposition of organic compounds. *Moraxellaceae* show a high tolerance to variable environmental conditions, including salinity, temperature, and the presence of pollutants, which makes them important components of the microflora of aquatic environments with different trophic characteristics. Together, both families of bacteria contribute to maintaining the biogeochemical balance in aquatic environments, including beaver ponds, by regulating the rate of organic matter decomposition and influencing the availability of nutrients for other aquatic organisms.

Analyses have shown that the most important indicator families in beaver pond sediments are *Planococcaceae* and *Desulfovibrionaceae*. The *Planococcaceae* family comprises species with very different morphological, physiological, and biochemical characteristics. The vast majority of taxa are typical of soil biota, but there are also species that occur in bottom sediments and surface waters (26). Some *Planococcaceae* species may be involved in the decomposition processes of organic matter in sediments, which can affect the chemical composition of sediments and biogeochemical cycling in the bottom environment. They may be present in bottom sediments, especially if these sediments are organically contaminated. Their presence in contaminated sediments also makes them important for environmental monitoring (26, 27). Bacteria from the *Desulfovibrionaceae* family play a key role in sediments as sulfate-reducing bacteria (28). Their presence is particularly important in anoxic sediment environments. They contribute to the decomposition of organic compounds, which leads to the production of hydrogen sulfide. This process can have an impact on metal contaminants, as the metal sulfides produced by these bacteria can bind heavy metals, which is important for bioremediation (29, 30).

Beaver pond ecosystems created by beaver activities (e.g., dam building) are characterized by unique conditions—water stagnation, increased sedimentation, accumulation of organic material and the formation of anaerobic zones. These changes favor the selection of certain groups of microorganisms, including anaerobic bacteria and eutrophying indicator bacteria. The identification of certain bacterial families under such conditions can therefore be a useful tool for monitoring water and sediment quality in such ecosystems.

In addition, the results obtained can have practical application in the assessment of ecosystem services provided by beaver ponds, such as water retention, self-purification, and carbon and nitrogen storage. Furthermore, these results support the argument for the protection of beavers as an engineer species whose activities promote microbial diversity and stabilize biogeochemical processes.

## Data Availability

The sequences have been deposited in the NCBI database under numbers: SAMN50085966 - SAMN50086005, (BioProject accession number - PRJNA1294181).

## SUPPLEMENTAL MATERIAL

Table S1 spectrum.01092-25-s0001.docx Diversity of family composition in water and sediment samples in terms of occurrence and mean number of relative abundance.

ASM does not own the copyrights to Supplemental Material that may be linked to, or accessed through, an article. The authors have granted ASM a non-exclusive, world-wide license to publish the Supplemental Material files. Please contact the corresponding author directly for reuse.

## References

[1] Rolauffs P, Hering D, Lohse S. 2001. Composition, invertebrate community and productivity of a beaver dam in comparison to other stream habitat types. Hydrobiologia 459:201–212. doi:10.1023/A:1012507613952

[2] Bush BM, Wissinger SA. 2016. Invertebrates in beaver-created wetlands and ponds, p 411–449. In Invertebrates in freshwater Wetlands

[3] Vorel JK, ed. 2016. Handbook for coexisting with beavers, p 1–137. Czech University of Life Sciences Prague, Prague.

[4] Feldhamer GA, Thompson BC, Chapman JA, eds. 2003. Beaver, p 288–310. In Wild mammals of north America: biology, management, and conservation, Second Edition. The Johns Hopkins University Press, Baker, B.W., Hill, E.P.

[5] Czech A. 2000. Bóbr. Wydawnictwo Lubuskiego Klubu Przyrodników, Świebodzin.

[6] Schwab G. 2009. Biber in Bayern, p 49. In Biologie und management. Bayerisches Landesamt für Umwelt, Germany.

[7] Campbell-Palmer R, Rosell F, eds. 2013. Captive management guidelines for Eurasian beavers (Castor fiber), p 104. The Royal Zoological Society of Scotland.

[8] Rozhkova-Timina IO, Popkov VK, Mitchell PJ, Kirpotin SN. 2018. Beavers as ecosystem engineers – a review of their positive and negative effects. IOP Conf Ser: Earth Environ Sci 201:012015. doi:10.1088/1755-1315/201/1/012015

[9] Collen P, Gibson RJ. 2000. The general ecology of beavers (Castor spp.), as related to their influence on stream ecosystems and riparian habitats, and the subsequent effects on fish – a review. Rev Fish Biol Fish 10:439–461. doi:10.1023/A:1012262217012

[10] McDowell DM, Naiman RJ. 1986. Structure and function of a benthic invertebrate stream community as influenced by beaver (Castor canadensis). Oecologia 68:481–489. doi:10.1007/BF00378759 28311700

[11] Songster-Alpin MS, Klotz RL. 1995. A comparison of electron transport system activity in stream and beaver pond sediments. Can J Fish Aquat Sci 52:1318–1326. doi:10.1139/f95-128

[12] Behera BK, Dehury B, Rout AK, Patra B, Mantri N, Chakraborty HJ, Sarkar DJ, Kaushik NK, Bansal V, Singh I, Das BK, Rao AR, Rai A. 2021. Metagenomics study in aquatic resource management: recent trends, applied methodologies and future needs. Gene Rep 25:101372. doi:10.1016/j.genrep.2021.101372

[13] Amann RI, Ludwig W, Schleifer KH. 1995. Phylogenetic identification and in situ detection of individual microbial cells without cultivation. Microbiol Rev 59:143–169. doi:10.1128/mr.59.1.143-169.1995 7535888 PMC239358

[14] Handelsman J, Rondon MR, Brady SF, Clardy J, Goodman RM. 1998. Molecular biological access to the chemistry of unknown soil microbes: a new frontier for natural products. Chem Biol 5:R245–R249. doi:10.1016/S1074-5521(98)90108-9 9818143

[15] Cabello-Yeves PJ, Callieri C, Picazo A, Mehrshad M, Haro-Moreno JM, Roda-Garcia JJ, Dzhembekova N, Slabakova V, Slabakova N, Moncheva S, Rodriguez-Valera F. 2013. The microbiome of the Black Sea water column analyzed by shotgun and genome centric metagenomics. Environmental Microbiome 16. doi:10.1186/s40793-021-00374-1 PMC8067304 33902743

[16] Nguyen SG, Raza S, Ta LT, Le L-A, Ho CT, Unno T. 2022. Metagenomic investigation of the seasonal distribution of bacterial community and antibiotic-resistant genes in Day River Downstream, Ninh Binh, Vietnam. Appl Biol Chem 65. doi:10.1186/s13765-022-00687-w

[17] Ngugi DK, Salcher MM, Andrei A-S, Ghai R, Klotz F, Chiriac M-C, Ionescu D, Büsing P, Grossart H-P, Xing P, Priscu JC, Alymkulov S, Pester M. 2023. Postglacial adaptations enabled colonization and quasi-clonal dispersal of ammonia-oxidizing archaea in modern European large lakes. Sci Adv 9:eadc9392. doi:10.1126/sciadv.adc9392 36724220 PMC9891703

[18] Bolyen E, Rideout JR, Dillon MR, Bokulich NA, Abnet CC, Al-Ghalith GA, Alexander H, Alm EJ, Arumugam M, Asnicar F, et al.. 2019. Reproducible, interactive, scalable and extensible microbiome data science using QIIME 2. Nat Biotechnol 37:852–857. doi:10.1038/s41587-019-0209-9 31341288 PMC7015180

[19] Dufrêne M, Legendre P. 1997. Species assemblages and indicator species: the need for a flexible asymmetrical approach. Ecol Monogr 67:345–366. doi:10.1890/0012-9615(1997)067[0345:SAAIST]2.0.CO;2

[20] Cáceres MD, Legendre P. 2009. Associations between species and groups of sites: indices and statistical inference. Ecology 90:3566–3574. doi:10.1890/08-1823.1 20120823

[21] R Core Team. 2023. R: a language and environment for statistical computing. R Foundation for Statistical Computing, Vienna, Austria. https://www.R-project.org.

[22] Newton RJ, Jones SE, Eiler A, McMahon KD, Bertilsson S. 2011. A guide to the natural history of freshwater lake bacteria. Microbiol Mol Biol Rev 75:14–49. doi:10.1128/MMBR.00028-10 21372319 PMC3063352

[23] Wang H, Zhu R, Zhang X, Li Y, Ni L, Xie P, Shen H. 2019. Abiotic environmental factors override phytoplankton succession in shaping both free-living and attached bacterial communities in a highland lake. AMB Expr 9:170. doi:10.1186/s13568-019-0889-z PMC6823470 31673822

[24] Zhang L, Xu M, Li X, Lu W, Li J. 2020. Sediment bacterial community structure under the influence of different domestic sewage types. J Microbiol Biotechnol 30:1355–1366. doi:10.4014/jmb.2004.04023 32627763 PMC9728189

[25] Rout AK, Tripathy PS, Dixit S, Behera DU, Behera B, Das BK, Behera BK. 2024. Metagenomics analysis of sediments of river Ganga, India for bacterial diversity, functional genomics, antibiotic resistant genes and virulence factors. Curr Res Biotechnol 7:100187. doi:10.1016/j.crbiot.2024.100187

[26] Li Y, Huang D, Sun W, Sun X, Yan G, Gao W, Lin H. 2022. Characterizing sediment bacterial community and identifying the biological indicators in a seawater-freshwater transition zone during the wet and dry seasons. Environ Sci Pollut Res 29:41219–41230. doi:10.1007/s11356-021-18053-6 35088267

[27] Yue Y, Tang Y, Cai L, Yang Z, Chen X, Ouyang Y, Dai J, Yang M. 2022. Co-occurrence relationship and stochastic processes affect sedimentary archaeal and bacterial community assembly in estuarine–coastal margins. Microorganisms 10:1339. doi:10.3390/microorganisms10071339 35889058 PMC9318014

[28] Kuever J. 2014. The family *Desulfovibrionaceae*. In Rosenberg E, DeLong EF, Lory S, Stackebrandt E, Thompson F (ed), The prokaryotes. Springer, Berlin, Heidelberg.

[29] Nakashima Y, Sonobe T, Hanada M, Kitano G, Sonoyama Y, Iwai K, Kimura T, Kusube M. 2024. Microbial detoxification of sediments underpins persistence of Zostera marina meadows. Int J Mol Sci 25:5442. doi:10.3390/ijms25105442 38791480 PMC11122150

[30] Park M-J, Kim YJ, Park M, Yu J, Namirimu T, Roh Y-R, Kwon KK. 2022. Establishment of genome based criteria for classification of the family Desulfovibrionaceae and proposal of two novel genera, Alkalidesulfovibrio gen. nov. and Salidesulfovibrio gen. nov. Front Microbiol 13:738205. doi:10.3389/fmicb.2022.738205 35694308 PMC9174804

