# Supplementary material for: Indicator bacteria in beaver ponds—research from Poland
Source: Microbiol Spectr. 2025 Nov 17;14(1):e01092-25. doi: 10.1128/spectrum.01092-25 (PMC12772270; doi:10.1128/spectrum.01092-25)
Supplement: Table S1 — Diversity of family composition in water and sediment samples in terms of occurrence and mean number of relative abundance. [file spectrum.01092-25-s0001.docx]

Table S1. Diversity of families composition in water and sediment samples in terms of occurrence and mean number of relative abundance. Family numbers (No.) consistent with Figure 2.

| No. | Family | Sediment | | | Water | | | Difference in occurrences between substrates |
| --- | --- | --- | --- | --- | --- | --- | --- | --- |
|  |  | N | M | SE | N | M | SE |  |
| 1 | *Peptostreptococcaceae* | 19 | 1,1373 | 0,2568 | 8 | 0,7687 | 0,3291 | 11 |
| 2 | *Sutterellaceae* | 8 | 0,0587 | 0,0268 | . | . | . | 8 |
| 3 | *ST-12K33* | 9 | 0,2252 | 0,1345 | 1 | 0,0020 | 0,0020 | 8 |
| 4 | *Oscillospirales* | 9 | 0,3007 | 0,1092 | 1 | 0,0477 | 0,0465 | 8 |
| 5 | *Williamwhitmaniaceae* | 19 | 2,5057 | 0,5631 | 12 | 1,4336 | 0,5824 | 7 |
| 6 | *Desulfovibrionaceae* | 12 | 0,1188 | 0,0487 | 5 | 0,0196 | 0,0101 | 7 |
| 7 | *Oscillospiraceae* | 9 | 1,1628 | 0,4683 | 2 | 0,1260 | 0,1046 | 7 |
| 8 | *Planococcaceae* | 19 | 16,2185 | 3,2953 | 13 | 3,0629 | 1,1488 | 6 |
| 9 | *Sporomusaceae* | 15 | 0,5437 | 0,4190 | 9 | 0,1005 | 0,0404 | 6 |
| 10 | *Ruminococcaceae* | 17 | 1,2927 | 0,3184 | 11 | 0,8845 | 0,2607 | 6 |
| 11 | *Burkholderiaceae* | 5 | 0,0829 | 0,0672 | . | . | . | 5 |
| 12 | *Lachnospiraceae* | 18 | 1,1408 | 0,2039 | 13 | 0,2732 | 0,1268 | 5 |
| 13 | *Family4* | 7 | 0,4097 | 0,3367 | 2 | 0,0444 | 0,0369 | 5 |
| 14 | *Anaerovoracaceae* | 11 | 0,2892 | 0,0979 | 7 | 0,1842 | 0,0851 | 4 |
| 15 | *Butyricicoccaceae* | 4 | 0,0087 | 0,0049 | . | . | . | 4 |
| 16 | *Coriobacteriales* | 5 | 0,0466 | 0,0297 | 1 | 0,0024 | 0,0024 | 4 |
| 17 | *Rhizobiaceae* | 5 | 0,4930 | 0,4167 | 1 | 0,0038 | 0,0037 | 4 |
| 18 | *uncultured2* | 3 | 0,0300 | 0,0164 | . | . | . | 3 |
| 19 | *Bacillaceae* | 12 | 0,0838 | 0,0332 | 9 | 0,1208 | 0,0882 | 3 |
| 20 | *Erysipelatoclostridiaceae* | 2 | 0,0030 | 0,0020 | . | . | . | 2 |
| 21 | *Paenibacillaceae* | 18 | 0,3648 | 0,0599 | 16 | 0,1257 | 0,0322 | 2 |
| 22 | *Alcaligenaceae* | 8 | 0,3756 | 0,1335 | 6 | 0,5796 | 0,2590 | 2 |
| 23 | *uncultured3* | 5 | 0,7646 | 0,6519 | 3 | 0,0088 | 0,0078 | 2 |
| 24 | *Family1* | 1 | 0,0001 | 0,0001 | . | . | . | 1 |
| 25 | *Micrococcaceae* | 1 | 0,0009 | 0,0009 | . | . | . | 1 |
| 26 | *Sulfurospirillaceae* | 1 | 0,0015 | 0,0015 | . | . | . | 1 |
| 27 | *Aneurinibacillaceae* | 1 | 0,0836 | 0,0815 | . | . | . | 1 |
| 28 | *Family3* | 1 | 0,0001 | 0,0001 | . | . | . | 1 |
| 29 | *Hungateiclostridiaceae* | 1 | 0,0038 | 0,0037 | . | . | . | 1 |
| 30 | *Peptococcaceae* | 1 | 0,0023 | 0,0023 | . | . | . | 1 |
| 31 | *Family5* | 1 | 0,0008 | 0,0008 | . | . | . | 1 |
| 32 | *Pseudoalteromonadaceae* | 1 | 0,0002 | 0,0002 | . | . | . | 1 |
| 33 | *Rhodocyclaceae* | 1 | 0,0023 | 0,0023 | . | . | . | 1 |
| 34 | *Pectobacteriaceae* | 1 | 0,0046 | 0,0045 | . | . | . | 1 |
| 35 | *Vibrionaceae* | 1 | 0,0002 | 0,0002 | . | . | . | 1 |
| 36 | *Rikenellaceae* | 4 | 0,0480 | 0,0247 | 3 | 0,0064 | 0,0053 | 1 |
| 37 | *Family2* | 2 | 0,0012 | 0,0011 | 1 | 0,0014 | 0,0014 | 1 |
| 38 | *Streptococcaceae* | 2 | 0,0040 | 0,0030 | 1 | 0,0029 | 0,0028 | 1 |
| 39 | *Peptostreptococcales-Tissierellales* | 2 | 0,2035 | 0,1979 | 1 | 0,0019 | 0,0019 | 1 |
| 40 | *Sedimentibacteraceae* | 2 | 0,0095 | 0,0073 | 1 | 0,0293 | 0,0285 | 1 |
| 41 | *Aeromonadaceae* | 20 | 6,9846 | 1,0544 | 20 | 8,4227 | 1,4275 | 0 |
| 42 | *Pseudomonadaceae* | 20 | 29,0570 | 4,5965 | 20 | 35,0708 | 3,7298 | 0 |
| 43 | *Selenomonadaceae* | 2 | 0,0018 | 0,0013 | 2 | 0,0073 | 0,0061 | 0 |
| 44 | *Eggerthellaceae* | 1 | 0,0029 | 0,0028 | 1 | 0,0020 | 0,0020 | 0 |
| 45 | *Prevotellaceae* | 1 | 0,0029 | 0,0029 | 1 | 0,0033 | 0,0032 | 0 |
| 46 | *Arcobacteraceae* | 1 | 0,0151 | 0,0147 | 1 | 0,0537 | 0,0523 | 0 |
| 47 | *Brevibacillaceae* | 1 | 0,0013 | 0,0013 | 1 | 0,0025 | 0,0025 | 0 |
| 48 | *Dysgonomonadaceae* | 19 | 1,5455 | 0,2664 | 20 | 1,7882 | 0,4503 | -1 |
| 49 | *Clostridiaceae* | 19 | 4,8548 | 0,6047 | 20 | 3,3566 | 0,8660 | -1 |
| 50 | *Shewanellaceae* | 16 | 1,3702 | 0,3701 | 17 | 1,9202 | 0,4097 | -1 |
| 51 | *Comamonadaceae* | 16 | 13,8407 | 3,4628 | 17 | 10,5072 | 2,1343 | -1 |
| 52 | *Orbaceae* | 2 | 0,0210 | 0,0141 | 3 | 0,0116 | 0,0075 | -1 |
| 53 | *Sphingobacteriaceae* | 1 | 0,0007 | 0,0007 | 2 | 0,0365 | 0,0353 | -1 |
| 54 | *Caloramatoraceae* | 1 | 0,0004 | 0,0004 | 2 | 0,0044 | 0,0037 | -1 |
| 55 | *Microbacteriaceae* | . | . | . | 1 | 0,0006 | 0,0006 | -1 |
| 56 | *Neisseriaceae* | . | . | . | 1 | 0,0087 | 0,0085 | -1 |
| 57 | *Acidaminococcaceae* | 10 | 0,3014 | 0,1324 | 11 | 0,7638 | 0,2495 | -1 |
| 58 | *Bacteroidaceae* | 18 | 3,0234 | 0,6857 | 20 | 3,7772 | 0,6953 | -2 |
| 59 | *Yersiniaceae* | 16 | 0,7673 | 0,2978 | 18 | 0,2795 | 0,0795 | -2 |
| 60 | *Tannerellaceae* | 15 | 2,0347 | 0,5241 | 17 | 1,9130 | 0,4723 | -2 |
| 61 | *Muribaculaceae* | 1 | 0,0033 | 0,0033 | 3 | 0,0045 | 0,0026 | -2 |
| 62 | *uncultured1* | . | . | . | 2 | 0,0021 | 0,0014 | -2 |
| 63 | *Alteromonadaceae* | . | . | . | 2 | 0,0136 | 0,0116 | -2 |
| 64 | *Budviciaceae* | 9 | 0,2598 | 0,1124 | 11 | 0,2273 | 0,0723 | -2 |
| 65 | *Enterobacteriaceae* | 13 | 0,6881 | 0,2730 | 16 | 1,0061 | 0,3341 | -3 |
| 66 | *Hafniaceae* | 10 | 0,6642 | 0,2355 | 13 | 1,1616 | 0,3090 | -3 |
| 67 | *Erysipelotrichaceae* | 9 | 0,0394 | 0,0202 | 12 | 0,1371 | 0,0492 | -3 |
| 68 | *Flavobacteriaceae* | 2 | 0,2258 | 0,2133 | 5 | 0,4127 | 0,2832 | -3 |
| 69 | *Vagococcaceae* | 1 | 0,0021 | 0,0020 | 4 | 0,0124 | 0,0075 | -3 |
| 70 | *Exiguobacteraceae* | 8 | 0,1396 | 0,0789 | 11 | 0,1402 | 0,0485 | -3 |
| 71 | *Chromobacteriaceae* | 12 | 2,3573 | 1,6485 | 16 | 5,4802 | 3,5652 | -4 |
| 72 | *Carnobacteriaceae* | 4 | 0,0135 | 0,0073 | 8 | 0,0530 | 0,0181 | -4 |
| 73 | *Aquaspirillaceae* | 3 | 1,4958 | 1,2043 | 8 | 3,4256 | 1,8625 | -5 |
| 74 | *Acidaminobacteraceae* | 1 | 0,0042 | 0,0041 | 6 | 0,6162 | 0,3375 | -5 |
| 75 | *Morganellaceae* | 1 | 0,1662 | 0,1620 | 6 | 0,2494 | 0,1114 | -5 |
| 76 | *Fusobacteriaceae* | 11 | 1,0507 | 0,4882 | 17 | 6,9935 | 1,7621 | -6 |
| 77 | *Enterococcaceae* | 7 | 0,0476 | 0,0233 | 13 | 0,3335 | 0,1614 | -6 |
| 78 | *Family6* | 4 | 0,0087 | 0,0047 | 10 | 0,0818 | 0,0304 | -6 |
| 79 | *Paludibacteraceae* | 3 | 0,0172 | 0,0117 | 10 | 0,1344 | 0,0734 | -7 |
| 80 | *Leptotrichiaceae* | 2 | 0,0019 | 0,0013 | 9 | 0,0112 | 0,0035 | -7 |
| 81 | *Caulobacteraceae* | 4 | 0,0046 | 0,0022 | 12 | 0,0495 | 0,0150 | -8 |
| 82 | *Moraxellaceae* | 4 | 0,0349 | 0,0217 | 12 | 1,0165 | 0,4630 | -8 |
| 83 | *Xanthomonadaceae* | 5 | 0,9270 | 0,8806 | 18 | 2,6783 | 0,9829 | -13 |

N – number of occurrences, M – arithmetic mean, SE – standard error
